# Supplementary figures and images for: Exploring the diagnostic potential of miRNA signatures in the Fabry disease serum: A comparative study of automated and manual sample isolations
Source: PLoS One. 2024 Oct 28;19(10):e0301733. doi: 10.1371/journal.pone.0301733 (PMC11515968; doi:10.1371/journal.pone.0301733)

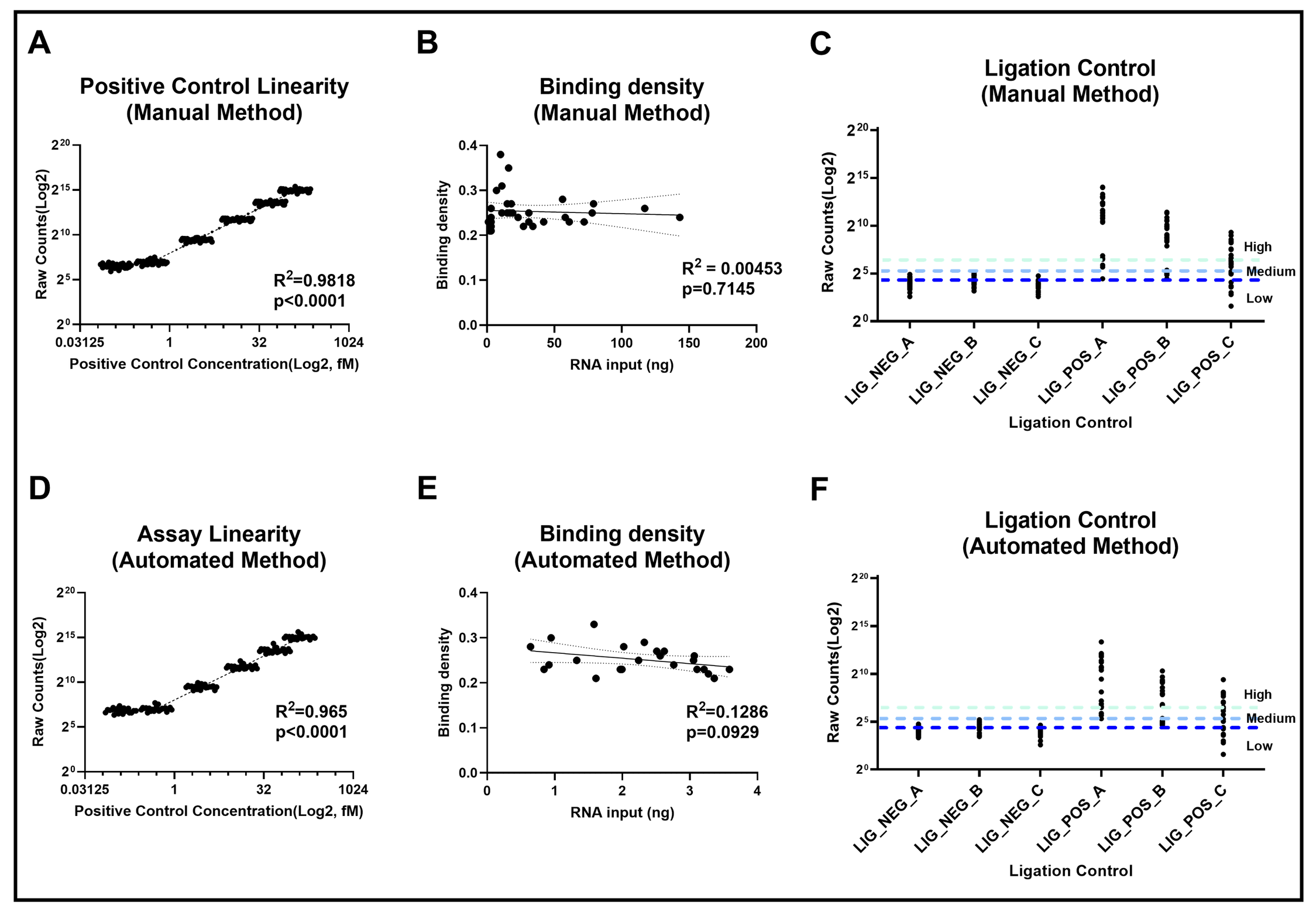

Supplement: S1 Fig — A and D) Correlation analysis between raw counts (Log2) and positive control concentration (Log2. fm). A Simple linear regression of R2 was calculated for the data. B and E) Correlation analysis between binding density and RNA input concentration(ng). Pearson correlation with linear regression analysis was performed. Mean linear regression is plotted (black straight line) with 95% confidence intervals (dashed line). C and F) Digital counts (Log2) are shown for all ligation controls. Average Low LOD, Medium LOD, and High LOD thresholds calculated for all negative controls are highlighted in shades of blue. (TIF) [file pone.0301733.s001.tif]

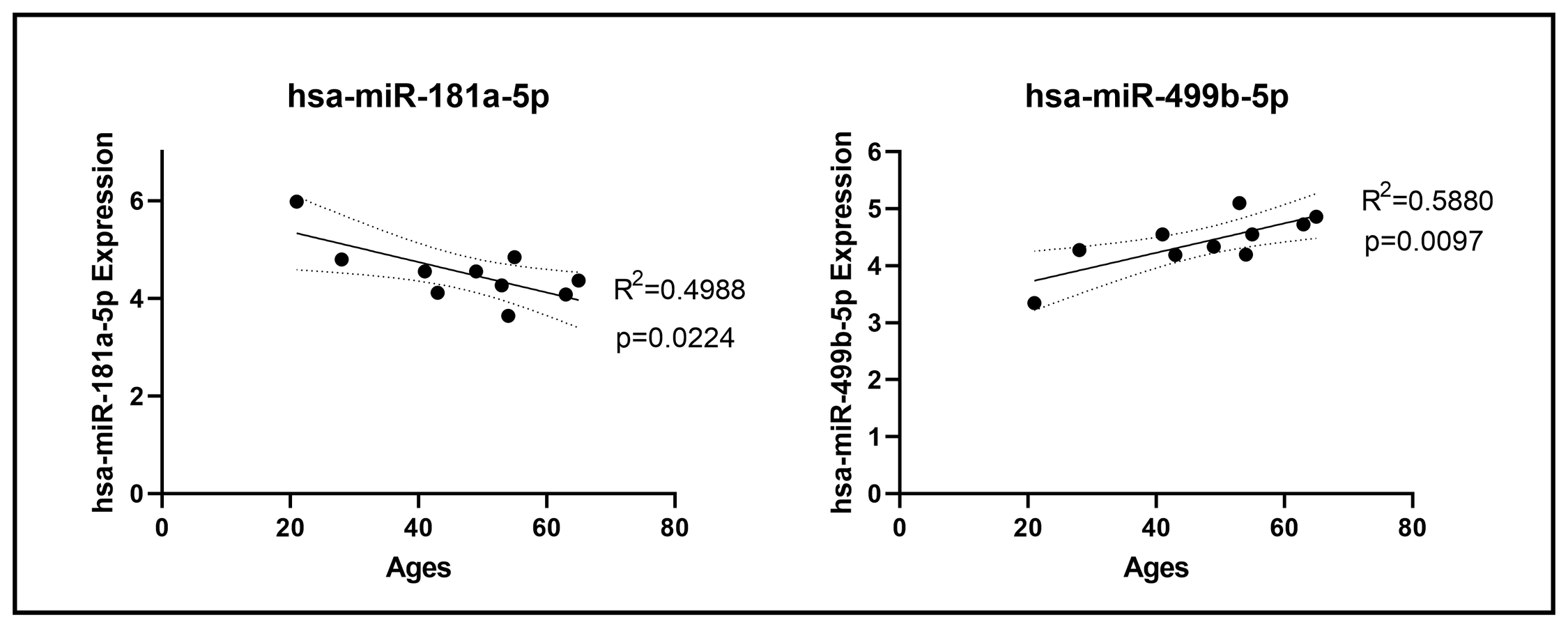

Supplement: S2 Fig — The association between age and miRNAs expression was performed by Person Correlation Coefficient. Both P values were lower than 0.05. (TIF) [file pone.0301733.s002.tif]
